# Auxin supplementation under nitrogen limitation enhanced oleic acid and MUFA content in *Eustigmatos calaminaris* biomass with potential for biodiesel production

**DOI:** 10.1038/s41598-023-27778-y

**Published:** 2023-01-11

**Authors:** Izabela Krzemińska, Marlena Szymańska, Wioleta Ciempiel, Agata Piasecka

**Affiliations:** Institute of Agrophysics, Polish Academy of Sciences, Doświadczalna 4, 20-290 Lublin, Poland

**Keywords:** Applied microbiology, Environmental microbiology, Environmental impact

## Abstract

Due to their lipid accumulation potential, microalgae are widely studied in terms of their use in the production of biodiesel. The present study was focused on determination of changes in the biomass production, biochemical composition, accumulation and distribution of fatty acids in neutral lipids, glycolipids, phospholipids and biodiesel properties of soil microalga *Eustigmatos calaminaris* in response to various levels of nitrogen stress and indole-3-acetic acid supplementation. The highest growth rate, the highest lipid content and daily lipid productivity were noted at the nitrogen limitation up to 25% with IAA supplementation. The increase in NL was associated with nutrient stress. An increase in the level of GL and PL were recorded upon the reduction of the nitrogen content (25% N) and the addition of IAA. The gas chromatography/mass spectrometry analysis demonstrated that C16:0, C16:1, and C18:1 were the main fatty acids in *E*. *calaminaris* lipids. As shown by the lipidomic analysis, the IAA supplementation in the nitrogen limitation variants enhanced the content of TAGs in C18:1 and monounsaturated fatty acids. The current findings indicated a potential strategy to improve the fatty acid profile in neutral lipids and high potential of *E. calaminaris* for biodiesel applications.

## Introduction

Given the ability to synthesise and accumulate lipids, and in particular triacylglycerides (TAGs), the biomass of unicellular algae is one of the promising substrates for biodiesel production^1^. Due to the various physiological functions of lipids, their content and composition in algal cells varies, depending on the species and environmental conditions. The following lipid classes are distinguished in their composition: neutral lipids (NL) and polar lipids, i.e. glycolipids (GL) and phospholipids (PL). The NL class is represented mainly by triacylglycerides serving as a reserve material in cells. GLs are the basic components of thylakoids, while PLs are components of cell membranes and organelles^2^. The rate of synthesis and accumulation of neutral lipids or structural polar lipids changes depending e.g. on the growth conditions. In favourable environmental conditions, algae synthesise lipids in the form of phospholipids in their cell membranes^3^. Stress factors present in the environment induce changes in the growth and metabolism of algal cells. As shown by the current literature, nitrogen deficiency promotes the accumulation of lipids^1,4^. Due to among others the formation of excess reactive oxygen species in stress conditions, the accumulation of lipids is accompanied by inhibition of algal cell division^4^. Auxins are plant hormones whose function is related to detoxification of reactive oxygen species^5^. The indole-3-acetic acid auxin has been reported to increase the growth and metabolism regulation in green algae^6–8^. The current literature provides data on the impact of indole-3-acetic acid in nitrogen-stress conditions on the growth and cellular composition of microalgae^9^. However, there is no detailed lipidomic information about changes induced in the fatty acid profile (including fatty acids in individual lipid classes with special consideration of neutral lipids) in response to nitrogen limitation and supplementation with exogenous indole-3-acetic acid. Noteworthy, the suitability of the raw material for biodiesel production is influenced not only by the amounts of individual lipid classes but also by the fatty acid profile in neutral lipids. Eustigmatophycean microalgae are considered a promising material for industrial applications due to their ability to produce fatty acids^10^. Some species belonging to the class Eustigmatophyceae, e.g. marine *Nannochloropsis* spp., are regarded as model oleaginous species and are being investigated extensively. In contrast, only few studies have been focused on *Eustigmatos* strains occurring in soil; additionally, the biotechnological potential of these algae has not been explored yet^11^. So far, Eustigmatophyceae algae of the genus *Nannochloropsis*, *Trachydiscusminutus, Vischeri* and *Eustigmatos magnus* have only been analysed in terms of their ability to synthesise lipid compounds^4,11–13^. The wide use of *Nannochloropsis spp.* in biotechnology suggests that also other Eustigmatophyceae species may be characterised by large biotechnological potential. *Eustigmatos calaminaris* (Eustigmatophyceae) is a unicellular coccoid algae isolated from calamine mine spoils, whose lipidomic profile has not yet been characterized so far^14,15^.

The study aimed at determination of the response of the non-model *Eustigmatos calaminaris* algae to different nitrogen limitation levels and indole-3-acetic acid supplementation in terms of biomass metabolic pathways and biochemical composition. To highlight the biotechnological potential of *E. calaminaris*, the current study presents new information about the biochemical composition and changes in the accumulation and distribution of fatty acids in individual lipid classes with special consideration of neutral lipids.

## Results

### Growth characteristics of *E. calaminaris*

**Figure 1:**
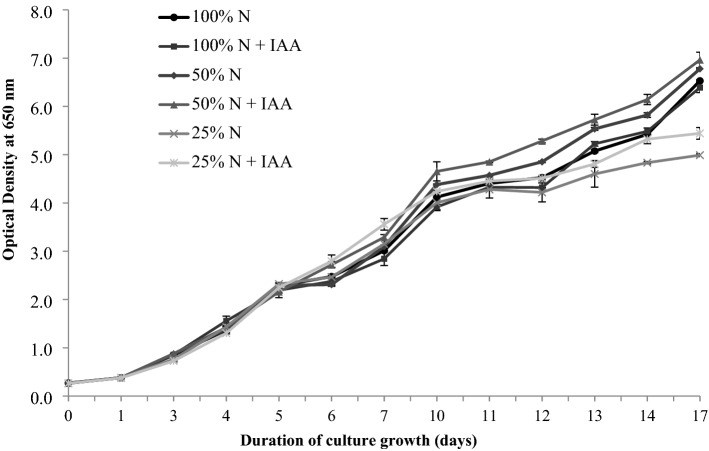
Growth curves of *Eustigmatos calaminaris* cultivated in: 100% NaNO_3_ (100% N), 100% NaNO_3_ and supplemented with IAA (100% N + IAA); 50% NaNO_3_ (50% N) and 50% NaNO_3_ supplemented with IAA (50% N + IAA); 25% NaNO_3_ (25% N) and 25% NaNO_3_ supplemented with IAA (25%N + IAA). Data are expressed as means (±SD), n = 9.

The *E. calaminaris* growth curves for all the experimental variants are shown in Fig. 1. The course of the growth curves is characterised by a short lag phase (day 0–1) followed by a logarithmic growth phase. The results showed no differences in the course of the *E. calaminaris* growth curves after the addition of IAA in the 100% N conditions, relative to the control. The effect of the IAA addition on the course of the growth curves, in comparison with the control, was visible after 5 days of the experiment in cultures grown in the 50% N and 25% N repletion conditions (Fig. 1).

The growth parameters in all the treatments are presented in Table 1. The specific growth rate observed in the IAA-supplemented cultures with 50% N and 25% N limitation was higher than in the control conditions. The highest specific growth rate value and the shortest *E. calaminaris* biomass doubling time were found in the nitrogen limitation conditions, i.e. at 25% N with the IAA supplementation: 0.374 µ day^−1^ and 44.27 h, respectively, in the cell growth period of 0–7 days. In the growth period of 0–10 days, the highest growth rate and the shortest biomass doubling time were observed in the 50% N+IAA variant: 0.286 µ day^−1^ and 57.87 h, respectively. In contrast, in the 100% N conditions, the IAA supplementation exerted no stimulating effect on the *E. calaminaris* growth rate.

**Table 1:** The influence of culture conditions on basis growth parameters of *Eustigmatos calaminaris*.

	100% N	100% N + IAA	50% N	50% N + IAA	25% N	25% N + IAA
Specific growth rate, 0–7 (day^−1^)	0.344 ± 0.0004a	0.342 ± 0.001a	0.350 ± 0.0005b	0.361 ± 0.0006c	0.354 ± 0.0032b	0.374 ± 0.0041d
Biomass doubling time 0–7 (h)	48.08 ± 0.06a	48.37 ± 0.15a	47.29 ± 0.07b	45.91 ± 0.07c	46.72 ± 0.43b	44.27 ± 0.49d
Specific growth rate, 0–10 (day^−1^)	0.273 ± 0.001a	0.267 ± 0.001b	0.281 ± 0.001c	0.286 ± 0.004d	0.270 ± 0.004ab	0.278 ± 0.001c
Biomass doubling time 0–10 (h)	60.60 ± 0.155a	62.04 ± 0.344b	58.83 ± 0.261c	57.85 ± 0.874d	61.26 ± 0.977ab	59.61 ± 0.114c
Biomass yield (g L^−1^)	3.27 ± 0.03a	3.20 ± 0.05a	3.59 ± 0.19b	3.67 ± 0.15b	2.23 ± 0.25c	2.94 ± 0.07d
Daily biomass productivity(g L^−1^ day^−1^)	0.192 ± 0.002a	0.188 ± 0.003a	0.215 ± 0.010b	0.216 ± 0.006b	0.131 ± 0.015c	0.173 ± 0.004d

Data are expressed as means ( ± SD), n = 9. Means followed by the same letter are not significantly different; Tukey HSD test, *p* < 0.05.

The biomass yield ranged from 2.23±0.25 gL^−1^ to 3.67±0.15 gL^−1^. The highest *E. calaminaris* biomass yield and daily biomass productivity were achieved in the 50% N+IAA variant. In turn, the lowest biomass yield was found in the 25% N conditions. The results showed that the IAA supplementation produced a significant increase in the biomass yield and daily biomass productivity only in the 25% N repletion conditions.

### Analysis of carbohydrate and lipid content

**Figure 2:**
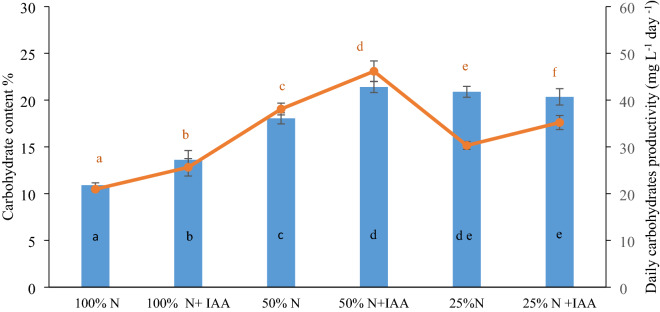
Impact of culture conditions on carbohydrate content (bars) and daily carbohydrates productivity (circles) in *Eustigmatos calaminaris.* Data are expressed as means (± SD), n = 9. Means followed by the same letter are not significantly different; Tukey HSD test, *p* < 0.05.

Figure 2 shows the percentage content of carbohydrates and the daily carbohydrate productivity (expressed in mg L^−1^) in the *E. calaminaris* cells. The addition of the plant hormone was found to contribute to a significant increase in the daily carbohydrate productivity of the *E. calaminaris* cells in each N availability variant, compared to the control conditions (with no IAA supplementation). The highest daily carbohydrate productivity of 46.146 (mg L^−1^ day^−1^) was observed in cells cultured in the 50% N conditions with the addition of IAA. The reduction of the nitrogen content to 25% N resulted in a decrease in the daily carbohydrate productivity in comparison with the 50% N conditions. The lowest daily carbohydrate productivity was obtained in the 100% N variant. The carbohydrate content ranged from 10.9 to 21.40% in the 100% N+IAA and 50% N+IAA conditions, respectively. It was found that the decrease in the nitrogen content in the medium from 100 N to 25% N was accompanied by an increase in the percent content of carbohydrates in the *E. calaminaris* cells. The IAA supplementation was found to induce carbohydrate accumulation in the cells in the 100% N and 50% N variants.

**Figure 3:**
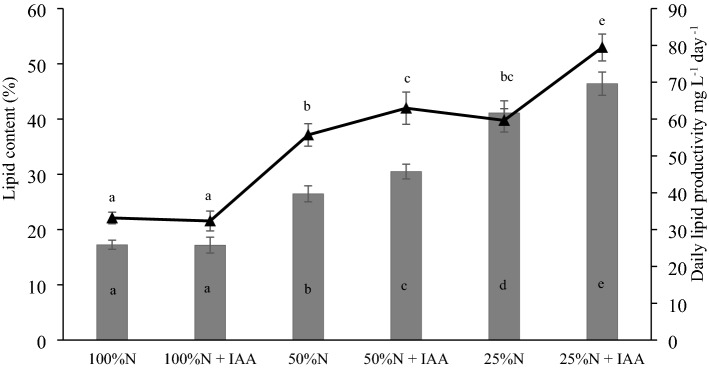
Impact of culture conditions on lipid content (gray bars) and daily lipid productivity (black triangles) in *Eustigmatos calaminaris*. Data are expressed as means (± SD), n = 9. Means followed by the same letter are not significantly different; Tukey HSD test, *p *< 0.05.

It was found that the lipid content in the *E. calaminaris* cells and the daily lipid productivity increased with the decreasing nitrogen content in the medium. As shown in Fig. 3, the supplementation with indole-acetic acid increased the lipid content and the daily lipid productivity in *E. calaminaris* cells in the 50% N and 25% N limitation conditions. The results showed that the IAA supplementation did not significantly alter the lipid content in the *E. calaminaris* cells in the 100% N conditions. The highest lipid content of 46.40% and the highest daily lipid productivity of 79.44 (mg L^−1^ day^−1^) were exhibited by the cells cultured in the 25% N limitation with IAA supplementation variant.

**Figure 4:**
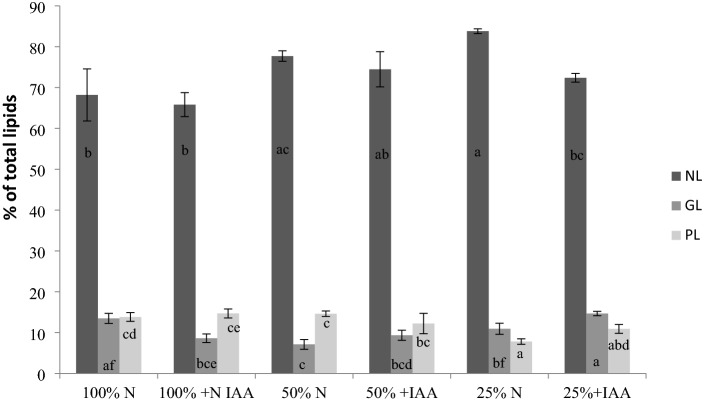
The influence of culture conditions on lipid class composition in *Eustigmatos calaminaris* cells. NL, neutral lipids; GL, glycolipids; PL, phospholipids. Data are expressed as means (± SD), n = 9. Means followed by the same letter are not significantly different; Tukey HSD test, *p* < 0.05.

Total lipids were fractionated to determine the synergistic effect of IAA and the different nitrogen levels on the lipid composition. Neutral lipids were the dominant lipid fraction in all the variants of the experiment. The study showed significant accumulation of NL in *E. calaminaris* in the conditions of the reduction of the nitrogen content to 25% N. The increase in the NL fraction was a response to the reduced nitrogen content (Fig. 4). The lowest content of neutral lipids was detected at the 100% N+ IAA conditions (66%). The reduction of the nitrogen content (25% N) and the addition of IAA resulted in a decrease in the content of the NL fraction and an increase in the content of GL and PL in *E. calaminaris*. Despite the decline induced by the IAA supplementation, the content of NL was higher than in the 100% N control. In response to the decrease in the N content from 100% to 50% N and from 100 to 25%, the content of the GL fraction decreased. The highest GL content was detected in cells cultured in the 25% N with the IAA addition variant, and the highest content of PL was determined in the N repletion conditions with IAA supplementation (Fig. 4).

### Lipidomic analysis

**Figure 5:**
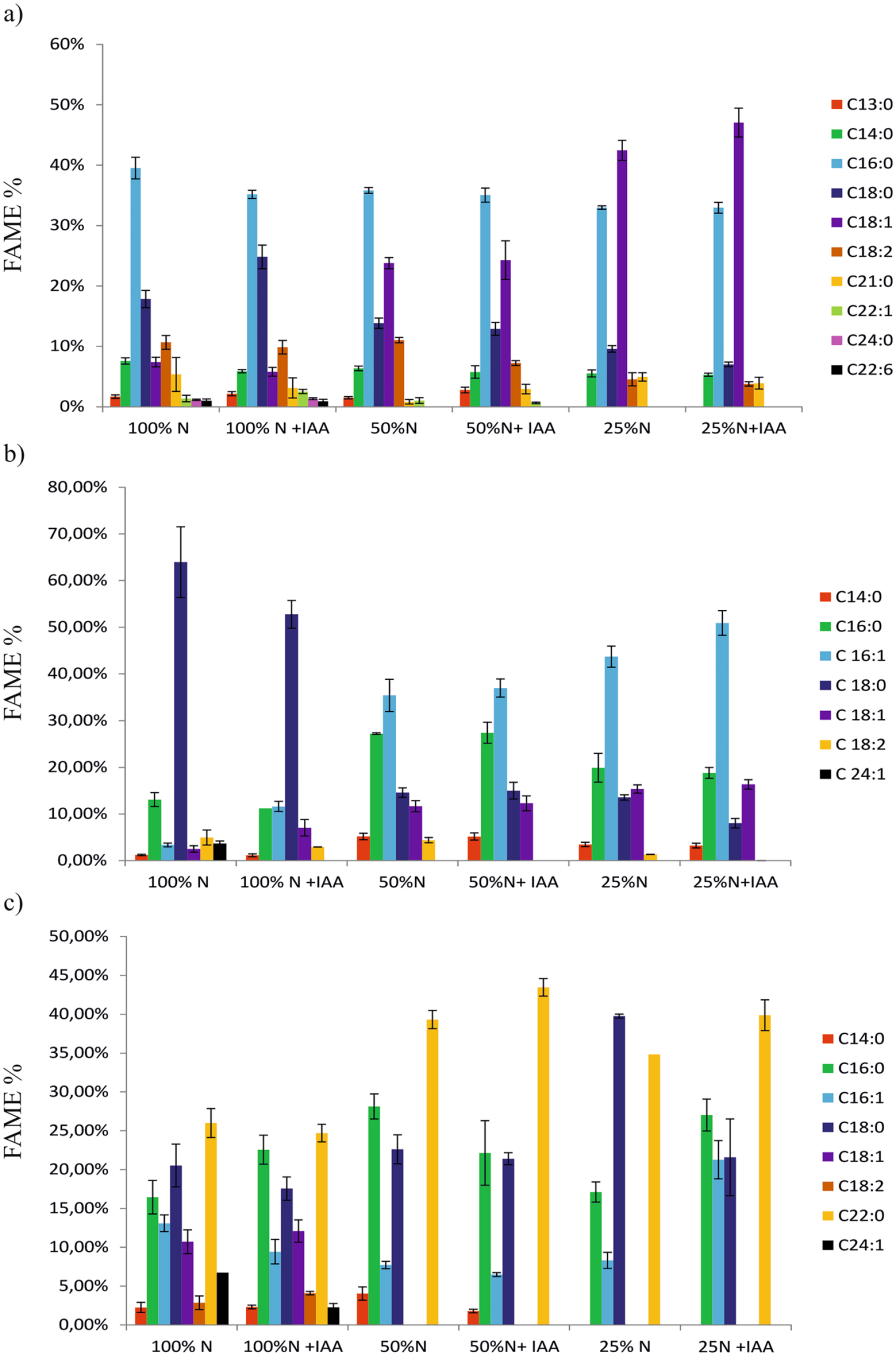
Effects of culture conditions on the fatty acid profile of *Eustigmatos calaminaris* (**a**) neutral lipids, (**b**) glycolipids, (**c**) phospholipids. Data are expressed as means (± SD), n = 9.

To obtain lipidomic information about the distribution of the fatty acid profile within the key lipid classes (NL, GL, and PL), *E. calaminaris* lipids were investigated using GC-MS. Figure 5 presents the characteristics of the fatty acid profile in *E. calaminaris.* Five major saturated fatty acids (over 2% of all fatty acids), i.e. myristic acid (C14:0), palmitic acid (C16:0), stearic acid (C18:0), heneicosylic acid (C21:0), and behenic acid (C22:0), were detected in all the culture variants. Unsaturated fatty acids (content exceeding 2%) were represented by monounsaturated acids: palmitoleic (C16:1) acid, oleic acid (C18:1), and nervonic acid (C24:1) and polyunsaturated linoleic acid (C18:2).

Oleic acid was the major acid in the neutral lipid fraction. The highest content of oleic acid was determined in the conditions of nitrogen limitation of 25% N with IAA supplementation. The decrease in the content of N in the medium was accompanied by an increase in the content of this acid in the NL fraction from 7.4 to 42.5%. It was found that the addition of IAA in the nitrogen limitation (25% N) variant caused an increase in the accumulation of oleic acid in the *E. calaminaris* cells. The content of palmitic acid (C16:0) in the NL fraction ranged from 33 to 39%. A decrease in the content of this acid was associated with the decrease in the N content in the culture medium. The IAA supplementation did not exert an effect on the C16:0 content in the nitrogen limitation conditions. The results indicated that, in the 100% N variant, the addition of IAA caused a decrease in the palmitic acid content from 39.5 to 35%.

Stearic acids (C18:0) was the third major acid in the NL fraction of *E. calaminaris* lipids. It was found that the content of this acid declined with the decrease in the nitrogen content. The addition of IAA to the medium in the nitrogen limitation (25% N) conditions reduced the content of this acid.

The GC-MS analysis indicated that palmitoleic acid (C16:1), stearic acid (C18:0), and palmitic acid (C16:0) were the main acids in the GL fraction in *E. calaminaris* lipids. It was found that the decrease in the N content in the medium was accompanied by accumulation of palmitoleic acid in the GL fraction. The addition of indole-3-acetic acid induced an increase in the C16:1 in the GL fraction. As can be seen in Fig. 5, the highest palmitoleic acid content (50.9%) was noted in the 25% N with IAA variant. In turn, the highest accumulation of stearic acid (62%) was recorded in the control (100% N). These results indicate that the IAA supplementation in the control conditions reduced the C18:0 content to 42%. Additionally, the content of stearic acid decreased together with the decrease in the nitrogen content in the culture medium.

The results indicated that the PL fraction of *E. calaminaris* comprised saturated fatty acids, i.e. palmitic (C16:0), stearic (C18:0), and behenic (C22:0) acids. Monounsaturated acids were represented mainly by the C16:1 acid and by the less abundant C24:1 acid. The presence of C18:1 was detected only in cells from cultures with 100% nitrogen availability in the medium. As in the case of the GL fraction, the IAA supplementation in the 25% N conditions induced an increase in the content of palmitoleic and palmitic acids and a decline in the content of stearic acid. Additionally, the content of stearic acid increased with the decrease in the N content in the medium.

Table 2 shows the sum of saturated fatty acids (SFAs), monounsaturated fatty acids (MUFAs), and polyunsaturated fatty acids (PUFAs). It was found that the SFA sum in the NL and GL fractions decreased with the decrease in the N content in the medium. The addition of IAA under nitrogen limitation to 25% reduced the SFA content in the NL, GL and PL fractions. As shown by the results, an increase in the total MUFA content in the NL, GL and PL fractions was observed in the variant with the reduced nitrogen content (25% N) in the medium and with the addition of IAA. *E. calaminaris* algae contain relatively low amounts of PUFAs. The total PUFA content in all fractions declined together with the reduction of the nitrogen content. The addition of IAA reduced the PUFA content in the NL fraction.

**Table 2.** The influence of culture conditions on the fatty acid profile of *Eustigmatos calaminaris.*

Fraction	Growth parameters	Fatty acid compositions (%)			
		Total C16–C18	SFA	MUFA	PUFA
NL	100% N	75.43 ± 3.02	73.11 ± 1.44	8.75 ± 1.32	6.33 ± 0.87
	100% N + IAA	75.63 ± 1.25	72.45 ± 1.14	8.31 ± 0.96	4.02 ± 1.28
	50% N	84.45 ± 0.54	58.30 ± 0.90	24.82 ± 1.42	0.82 ± 0.87
	50% N + IAA	79.46 ± 1.35	59.38 ± 2.42	24.90 ± 3.24	2.92 ± 0.69
	25% N	89.55 ± 0.66	53.00 ± 1.13	42.46 ± 1.94	4.93 ± 0.03
	25% N + IAA	90.80 ± 1.17	49.16 ± 2.34	47.05 ± 2.62	3.90 ± 0.48
GL	100% N	87.91 ± 4.63	78.28 ± 8.61	9.56 ± 1.44	4.96 ± 0.06
	100% N + IAA	85.59 ± 3.31	65.16 ± 2.73	18.67 ± 3.18	2.93 ± 0.02
	50% N	93.31 ± 4.25	47.02 ± 8.39	47.09 ± 1.96	4.39 ± 0.72
	50% N + IAA	91.69 ± 1.65	47.61 ± 2.73	49.26 ± 2.72	absence
	25% N	93.89 ± 0.47	36.94 ± 0.37	59.06 ± 7.13	1.35 ± 0.04
	25% N + IAA	94.13 ± 2.09	30.10 ± 2.27	67.27 ± 2.18	absence
PL	100% N	63.64 ± 5.58	65.22 ± 4,32	30.53 ± 3.91	2.85 ± 0,32
	100% N + IAA	65.75 ± 5.36	67.13 ± 4.30	23.80 ± 4.61	4.10 ± 0,20
	50% N	58.47 ± 5.59	94.12 ± 1.17	7.72 ± 0.85	absence
	50% N + IAA	50.03 ± 5.88	88.81 ± 3.76	6.48 ± 2.43	absence
	25% N	65.19 ± 2.60	91.69 ± 1.05	8.31 ± 0.37	absence
	25% N + IAA	69.88 ± 2.26	72.21 ± 1.62	21.28 ± 7.30	absence

Data are expressed as means ( ± SD), n = 9. *NL* neutral lipids, *GL* glycolipids, *PL* phospholipids. SFA–saturated fatty acids, MUFA–monounsaturated fatty acids, PUFA–polyunsaturated fatty acids.

The C16-C18 fatty acids in the NL fraction represented from 75.43 to 90.80% of all detected fatty acids. The highest amounts of C16-C18 fatty acids were observed in the 25% N+IAA conditions.

### Biodiesel evaluation

The parameters of the biodiesel quality were assessed in relation to the molecular structure of fatty acid methyl esters, which exhibits differences depending on the number and position of double bonds and depending on the carbon chain^16^. The following parameters of the NL fraction were determined to estimate the quality of biodiesel: cetane number (CN), long chain saturated factor (LCSF), and cold filter plugging point (CFPP) (Table 3). The estimated CN was similar in all experiment variants, with an average value of 55.95. The highest LCSF and CFPP values were recorded in the 100% N and 100% N+IAA variants, whereas the lowest values were noted in 25% N and 25% N+IAA.

**Table 3:** Estimated properties of biodiesel: cetane number (CN), long chain saturated factor assessment (LCSF), cold-filter plugging point (CFPP).

Growth parameters	Biodiesel parameters		
	CN	LCSF (wt.%)	CFPP (°C)
Fraction NL			
100% N	55.955 ± 0.347	15.21 ± 1.19	27.44 ± 0.72
100% N + IAA	55.963 ± 0.365	18.45 ± 0.47	41.49 ± 1.46
50% N	55.944 ± 0.189	10.49 ± 0.73	16.49 ± 2.29
50% N + IAA	55.947 ± 0.494	9.72 ± 0.84	13.09 ± 2.60
25% N	55.939 ± 0.190	8.09 ± 0.25	8.93 ± 0.80
25% N + IAA	55.937 ± 0.241	4.37 ± 0.12	− 2.5 ± 0.38

The values are presented as means  ±  standard deviation (SD) (n = 3).

## Discussion

The present results have shown that the addition of IAA to the medium under nitrogen limitation plays a role in *E. calaminaris* growth and metabolic processes. The supplementation was found to support the growth of *E. calaminaris* in the 50% N and 25% N limitation conditions. The differences in the growth rates in the periods of 0–7 days and 0–10 days in the conditions of nitrogen limitation in the medium (25% N and 50% N) indicate that nitrogen was utilised by the cells in the 25% N culture after 7 days, which resulted in a slower cell growth rate. Similar to the study conducted by Jusoh et al., there was no effect of the addition of IAA in the 100% N variant^17^. This may have been related to the insufficient IAA concentration to promote cell division in the conditions of full nitrogen availability and an increased number of cells^17^. During the first 5 days of the culture, higher OD_650_ values were recorded in the 100% N variant than in the cell culture carried out under nitrogen limitation. The addition of IAA in the 25% N limitation variant resulted in a significant increase in the biomass yield and the daily biomass productivity. As reported in the current literature, depending on the concentrations of IAA and morphological and physiological traits of species, auxin may promote microalgal growth, exert no effect on growth processes, or have an inhibitory effect on the growth of unicellular algae^6,16,18^. IAA has been shown to alleviate the harmful effects of N stress^9^. The present study showed that the addition of an appropriate dose of IAA in the nitrogen limitation conditions stimulated *E. calaminaris* growth and biomass yield. The availability of nitrogen in the medium had an impact on the *E. calaminaris* growth parameters as well. In the conditions of nitrogen limitation in the culture medium (25%), the *E. calaminaris* cell proliferation was lower at the expense of intracellular nitrate derived from the gradual degradation of chlorophyll^19^, ribosomes, membrane-bound proteins, and some enzymes. e.g. ribulose-1,5-bisphosphate carboxylase/oxygenase^20^. However, the intracellular nitrogen source is insufficient to sustain cell growth for a longer time^19^. The lower values of the specific growth rate (0–10 days), biomass yield, and daily biomass productivity (determined after the termination of the culture) of cells growing in the 25% N conditions, compared to the 50% N and 100% N cultures, confirm this information.

It was found in the present study that the carbohydrate content and the daily carbohydrate productivity of *E. calaminaris* were influenced by the IAA supplementation. IAA increased the daily carbohydrate productivity in all the nitrogen availability variants. However, an increase in the carbohydrate content was observed only in the variants of 100% N and 50% N with IAA supplementation. Studies on *C. vulgaris* and *C. sorokiniana* have demonstrated that exogenously applied indole-3-acetic acid increases the content of monosaccharides in cells and produces an increase in the chlorophyll content in a concentration-dependent manner^9,19^. As shown by previous reports, auxins can stimulate photosynthetic activity by increasing the chlorophyll content, which may increase carbohydrate synthesis^21^. It has also been suggested that IAA applied in the conditions of reduced nitrate concentrations decreases the chlorophyll content in cells^9^. The present study showed a decrease in the intracellular carbohydrate content in the 25% N limitation variant. Although the N limitation conditions (25% N) were found to inhibit cell proliferation, they contributed to increased lipid accumulation in the *E. calaminaris* cells. In comparison to the control culture, the accumulation increased by 23.8% in the 25% N culture and by 29% in the 25% N+IAA variant. These findings suggest that the *E. calaminaris* strategy in overcoming nutrient stress in the presence of IAA consists in enhanced accumulation of lipids. Positive effects of IAA on lipid accumulation have also been evidenced in studies on *S. quadricauda* and *Ch. sorokiniana*^9,18,22^. In turn, the 50% N limitation conditions with the IAA supplementation led to enhanced accumulation of both carbohydrates and lipids in the *E*. *calaminaris* cells.

The reduction of the nitrogen content in the medium to 25% N induced an increase in the content of neutral lipids in the *E. calaminaris* cells. The increase in the NL fraction observed in the present study is in line with the findings reported by Pancha et al.^23^, who found a significant increase in the NL content in *Scenedesmus* sp. after 3 days of nitrogen starvation. Neutral lipids serve no structural function; they are represented mainly by triacylglycerols (TAGs) accumulated as storage compounds in lipid bodies in the cytoplasm^2^. The TAG function is not limited to the storage function only, as they also take part in the processes of adaptation to environmental conditions^24^. The accumulation of the NL fraction, regarded as a response to higher oxidative stress induced by environmental stress, is targeted at reduction of intracellular oxidative damage and prevention of cells from premature senescence^16^. TAGs are regarded as a suitable source of fatty acids for the production of biodiesel^25^. A study on *Nannochloropsis* sp. demonstrated an increase in the content of NL and a decrease in the content of GL and PL under reduced nitrogen availability^1^. Available literature reports show that 3-day nitrogen starvation induced a decline in the NL content in *Acutodesmus dimorphus* lipids^26^.

Palmitic acid (C16:0), palmitoleic acid (C16:1), and oleic acids (C18:1), which are characteristic of eustigmatophyceans^27^, were found to be the main fatty acids in the *E. calaminaris* fatty acid profile. The results obtained in the current study showed that, in the conditions of a reduced amount of N in the medium (25% N), IAA had an impact on the accumulation of olecic, palmitic, and palmitolecic acids. There are discrepant literature reports on the influence of IAA on the accumulation and profile of fatty acids in microalgal cells. Some reports suggest that IAA exerts an effect on the induction of C18:1 in N limitation conditions^9^. Increased amounts of palmitic and oleic acids in *S. quadricauda*cells were found in the presence of IAA^18^. In contrast, Park et al.^7^ demonstrated no impact of IAA on fatty acids in *C. reinhardtii.* An increase in the content of oleic acid in neutral lipids and an increase in the amounts of palmitoleic acid in glycolipids were also found to accompany a decrease in the nitrogen content. Previous research suggested that the increase in the content of C18:1 acids is associated with de novo synthesis of these acids and not only to the degradation of polar lipids (GL and PL)^25,28^. This biosynthesis can take place at the expense of energy derived from degradation of carbohydrates^28^, which explains the decrease in the carbohydrate content in *E. calaminaris* cells cultured in the 25% N conditions. Concurrently, high palmitic acid content in the NL fraction was detected in the nitrogen and IAA addition conditions. Palmitic and oleic acids are regarded as the most common components of biodiesel^29^. Due to their properties, monounsaturated fatty acids are preferred in the production of biodiesel. The increase in MUFAs in the neutral lipids of *E. calaminaris* observed in this study was similarly associated with the reduction in nitrogen content and the addition of IAA.

The fatty acid profile in *E. calaminaris* cultured in the nitrogen limitation and IAA addition conditions was dominated by high oleic acid content in TAGs and exhibited an increase in the MUFA content, which are beneficial for biodiesel application. Importantly, no growth inhibition was observed in the cultures.

Regardless of the growing conditions, the lipids produced by *E. calaminaris* were characterized by a stable cetane number: 55.95 (Table 3). The minimum CN value for high-quality biodiesel according to European standard EN 14214:2008 is 51. The CN values estimated for biodiesel from microalgal oil ranged from 42.47 to 64.94^16,30^. A higher cetane number results in a shorter ignition time ^16^ The CFPP parameter is used to predict biodiesel flow efficiency at low temperatures^30^ Higher CFPP values indicate worse low-temperature properties of biodiesel^30^. A decline in the CFPP value was observed along with the decrease in the N content in the cultures. The decrease in the CFPP value was also caused by the addition of IAA but only in the culture variants with reduced nitrogen content (50%N and 25%N). The lowest and most favourable value of the cold filter plugging point parameter (− 2.5°C) was exhibited by lipids from cultures with the nitrogen content reduced to 25% and the IAA supplementation. The low value of the CFPP parameter in the nutrient limitation conditions was related to the absence of lignoceric acid (C24:0) methyl esters and the lower content of palmitic acid methyl esters, compared to the control conditions (Fig. 5). Biodiesel containing high levels of stearic and palmitic acid ME has a high CFPP value. The study results indicate that the *E. calaminaris* lipids meet the requirements of the European standard EN 14214 for CN. Additionally, the nutrient limitation and IAA supplementation in these conditions had a beneficial effect on the value of the cold filter plugging point of the biodiesel.

## Materials and methods

### Microalgal strains

*Eustigmatos calaminaris* was purchased from the culture collection of autotrophic organisms (CCALA, TŘEBOŇ, Czech Republic). *Eustigmatos calaminaris* inocula were maintained in sterile media BG 11 with a soil extract. The soil extract was prepared in accordance with the recommendations from the from the culture collection of autotrophic organisms (Czech Republic).The microalga was grown in shaken Erlenmeyer flasks in the following conditions: 80 µmol photons m^−2^ s^−1^, light/dark cycle 18/6 h, and aeration with sterile air. The temperature was adjusted to 22 ± 1 °C.

### Batch culture experiments

The experiment was carried out in 200 ml of sterilized BG 11 (with 5 ml L^−1^ of the soil extract) in a 500 ml Erlenmeyer flask. The final concentration of IAA in the culture media was 10^−4^ M. The final concentration of IAA in the culture media was 10^−4^ M. The IAA concentration used in the experiments was selected based on previous studies^6^. The stock solution of indole 3-acetic acid was prepared using 98% ethanol, and the ethanol concentration in the medium did not exceed 1% v/v^18^. The BG11 medium supplemented with IAA was modified in terms of the content of the nitrogen source: 100% N of NaNO_3_ (100% N+IAA), 50% of NaNO_3_ (50%N+IAA) and 25% of NaNO_3_ (25%N+IAA), i.e. 1.5 gL^−1^, 0.75 gL^−1^, and 0.375 gL^−1^ of NaNO_3_, respectively_._ Cells grown in BG 11 without IAA additive (100%N, 50% N and 25% N) were used as a control culture.

### Assessment of microalgal growth

Microalgae were grown in a growth chamber under 80 µmol photons m^−2^ s^−1^ light intensity and a 16 h/8 h light/dark cycle, with orbital shaking at 100 rpm and aeration with sterile air. The growth temperature was 22 ± 1 °C. The cells were grown for 17 days.

Biomass accumulation was determined by measuring daily changes in optical density OD_650_ (Cary 300/Biomelt spectrophotometer). The specific growth rate µ (d^−1^) was calculated as follows:

1 $$\upmu = {\text{ ln}}\left( {{\text{N}}_{{2}} /{\text{N}}_{{1}} } \right)/\left( {{\text{T}}_{{2}} {-}{\text{ T}}_{{1}} } \right)$$

where N_1_ is the initial biomass concentration at time T_1_ and N_2_ is the biomass concentration at time T_2_^11^.

The doubling time of *E*. *calaminaris* biomass was calculated using Eq. (2):

2 $${\text{Td }}\left( {{\text{hr}}} \right) \, = \, \left( {{\text{ln2}}/\upmu } \right) \times {24}$$

### Assessment of biomass productivity

The biomass productivity (Bp) of *E. calaminaris* was determined gravimetrically. Algal cells were filtered through filters (Whatman GF/C) and dried to constant weight and weighed. The biomass productivity of *E. calaminaris* was calculated using the following equation:

3 $${\text{Bp }} = {\text{ DCW}}/{\text{T}}$$

where DCW- dry cell weight (g L^−1^ g mL^−1^),and t (day)—duration of the culture in days. The biomass productivity was expressed in g mL^−1^ day^−1^.

### Carbohydrate content

The total content of simple sugars was determined with the anthrone colorimetric method^31^. Briefly, a 2% anthrone solution in 98% H_2_SO_4_ was added to the samples, which were then heated in a boiling-water bath. The absorbance of the samples was measured at 620 nm (Cary 300/Biomelt). The content of sugars (mg ml^−1^) was determined with the use of the calibration curve prepared for the glucose standard. The content of carbohydrates was expressed in % (m/m).

### Extraction of crude lipids

Lipids were extracted from the centrifuged biomass after 17 days with a modified Bligh and Dyer method^11^. Briefly, the *E.calaminaris* biomass was subjected to a mixture of chloroform:methanol (1:2, v/v) with sonication. After evaporation of the solvents, the lipid content was determined gravimetrically. Butylated hydroxytoluene was used in the extraction process as an antioxidant. To determine the lipid content gravimetrically, the solvent was evaporated on a vacuum evaporator. Three replicates of each extraction were performed. Samples of extracted cellular lipids were suspended in n-hexane.

Lipid productivity (Lp) was calculated using the following Eq. (4):

4 $${\text{Lp }} = {\text{Bp }} \times {\text{Lc}}$$

where Bp—biomass productivity, Lc—lipid content. Lipid productivity was expressed in milligrams per litre per day.

### Lipid separation by solid phase extraction

The lipid extract was separatedusing silica cartridges BAKERBOND spe™ SiOH, 500 mg, as in Meng et al.^1^. The SPE cartridge had been conditioned with chloroform. Next, the lipid extract was applied on the SPE column. The cartridge was eluted with 10 mL of chloroform to collect neutral lipids (NL). Next, 10 mL of acetone was added to collect glycolipids (GL.) The phospholipids (PL) were obtained by elution with acetone and10 mL methanol. The eluent was dried under a nitrogen stream. The NL, GL, and PL contents were determined gravimetrically, after drying under a stream of nitrogen. The fractionated lipid classes were suspended in chloroform for further analysis.

### Fatty acid methyl ester formation

Fatty acids were converted to fatty acid methyl esters (FAMEs) as previously described by Krzemińska et al.^11^. Briefly, the analyses was performed in the following steps: the samples of fractionated lipid classes were supplemented with 0.5 M KOH in HPLC grade methanol, mixed, and hydrolyzed at 85–90 °C for 1 h. Next, 10% BF3 in 100% methanol was added to cooled samples and esterification was performed at 100 °C for 20 min. In the subsequent step, 99% n-hexane and a saturated NaCl solution were added.

### Determination of fatty acid methyl ester content

FAME analyses were performed using a Trace GC Ultra (Thermo Scientific) chromatograph coupled with a ITQ 1100 mass spectrometer (Thermo Scientific) with a capillary column Zebron ZB- FAME (dimensions: 60 m × 0.25 mm × 0.20 μm). Heptadecanoic acid was used as an internal standard. The chromatographic conditions were set as a follows: 4 min at 100 °C followed by heating to 140 °C at 10 °C/min, and next to190 °C at the rate of 2 °C/min, and to 260 °C at the rate of 30 °C/min for 5 min; sample injection volume of 2 µl, split ratio of 20:1, injection temperature of 250 °C. Helium was used as the carrier gas at a flow rate of 2.1 mL/min. The FAME MIX -37 (CRM 47885 Supelco) was used for identification of FAMEs. The fatty acid concentration and compositions were calculated from the area of the peaks, the internal standard concentration, and the amount of the sample. Two replicates were performed for each GCMS analysis.

### Statistical analysis of results

The experiments were carried out in three replications. Statistical analysis were carried out in the STATISTICA v. 13 program (TIBCO *Software* Inc. USA). Differences in the measured parameters were compared using two-way *ANOVA*, at a significance level of *p *≤ 0.05 using the post-hoc Tukey test.

### Estimation of biodiesel properties

The selected properties of the biodiesel from *E. calaminaris* neutral lipids were calculated using fatty acid methyl esters and based on equations (5–7) described by Afifudeen et al.^16^. The cetane number (CN) was calculated using the equation below:

5 $$\begin{aligned} {\text{CN}} & = {55}.{87} + \, 0.0{\text{747x}}^{{1}} + \, 0.0{\text{98x}}^{{2}} + \, 0.{\text{164x}}^{{3}} + \, 0.{\text{176x}}^{{4}} \\ &\quad - 0.0{5}0{\text{x}}^{{5}} + \, 0.00{\text{1x}}^{{6}} - 0.{14}0{\text{x}}^{{7}} - 0.{\text{273x}}^{{8}} \\ \end{aligned}$$

where, x is the percentage composition of fatty acid methyl esters (x^1^:C12:0; x^2^:C14:0; x^3^:C16:0; x^4^:C18:0; x^5^:C16:1; x^6^:C18:1; x^7^:C18:2; x^8^:C18:3).

The long chain saturated factor (LCSF) and the cold filter plugging point (CFPP) were calculated using the equations below:

6 $${\text{LCSF }} = \, \left( {0.{6 } \times {\text{ C16}}} \right) \, + \, \left( {0.{5 } \times {\text{ C18}}} \right) \, + \, \left( {{1 } \times {\text{ C2}}0} \right) \, + \, \left( {{1}.{5 } \times {\text{ C22}}} \right) \, + \, \left( {{2 } \times {\text{ C24}}} \right)$$

7 $${\text{CFPP }} = \, \left( {{3}.{1417 } \times {\text{ LCSF}}} \right) - {16}.{477}$$

## Data Availability

All data generated or analyzed during this study are included in this manuscript.
